# Supplementary material for: Characteristics and driving mechanisms of vegetation phenology variations in the Bosten Lake Basin, Xinjiang, China
Source: PLoS One. 2025 Sep 25;20(9):e0332896. doi: 10.1371/journal.pone.0332896 (PMC12463244; doi:10.1371/journal.pone.0332896)
Supplement: Attachment — Submitted filename: Modify the description.docx [file pone.0332896.s002.docx]

**Dear Editor,**

We sincerely appreciate the editor's efforts in reviewing our manuscript (ID:PONE-D-25-17547R1). We have addressed the following issues in response to your request and suggestion. Below I will answer the questions posed by the editor.

1. We note your current Data Availability statement is: “the data are all contained within the manuscript. Tick here if the URLs/accession numbers/DOIs will be available only after acceptance of the manuscript for publication so that we can ensure their inclusion before publication.” When completing the data availability statement of the submission form, you indicated that you will make your data available on acceptance. We strongly recommend all authors decide on a data sharing plan before acceptance, as the process can be lengthy and hold up publication timelines. Please note that, though access restrictions are acceptable now, your entire data will need to be made freely accessible if your manuscript is accepted for publication. This policy applies to all data except where public deposition would breach compliance with the protocol approved by your research ethics board. If you are unable to adhere to our open data policy, please kindly revise your statement to explain your reasoning and we will seek the editor's input on an exemption. Please be assured that, once you have provided your new statement, the assessment of your exemption will not hold up the peer review process.

**Reply:** Thank you for your opinions and suggestions. The data generated by the authors to support the results of this study is stored in a public repository. The data can be accessed at: <https://github.com/Likunyu7/dataC>.

2. Can you please upload an additional copy of your revised manuscript that does not contain any tracked changes or highlighting as your main article file. This will be used in the production process if your manuscript is accepted.  Please amend the file type for the file showing your changes to Revised Manuscript w/tracked changes. Please follow this link for more information: [http://blogs.PLOS.org/everyone/2011/05/10/how-to-submit-your-revised-manuscript/](http://blogs.plos.org/everyone/2011/05/10/how-to-submit-your-revised-manuscript/).

**Reply:** We have uploaded the revised manuscript without tracked changes or highlighting as the main article file.

3. Please provide the link to your map sources so we may check the licensing information for ourselves. This way we can assure compliancy with our copyright policy.

**Reply:** We appreciate the opinions and suggestions of the editors. The boundary figure of Xinjiang Uygur Autonomous Region, China, is from the Map World Service Center of the Department of Natural Resources of the People's Republic of China ([https://cloudcenter.tianditu.gov.cn](https://cloudcenter.tianditu.gov.cn/)). The boundary of the study area is based on the Google Earth Engine platform ([https://earthengine.google.com/](https://earthengine.google.com/)). The downloaded DEM data is extracted by using the river basin extraction and analysis method and does not require data access permission. All other data sources have been explained and linked in the manuscript. All images comply with the CC BY 4.0 license requirements of PLOS ONE.

We would like to extend our sincere appreciation to the editors for their dedicated efforts. We trust that the revisions made will meet with approval. We are truly grateful for your valuable comments and suggestions, which have been of great significance in enhancing the quality of our manuscript.

Sincerely yours,

Xuemei Wang

E-mail: [xmwang2022@xjnu.edu.cn](mailto:xmwang2022@xjnu.edu.cn)
